# Anti-Adhesive Activities of Flavan-3-ols and Proanthocyanidins in the Interaction of Group A-Streptococci and Human Epithelial Cells 

**DOI:** 10.3390/molecules15107139

**Published:** 2010-10-15

**Authors:** Aneta Janecki, Herbert Kolodziej

**Affiliations:** Institute of Pharmacy, Pharmaceutical Biology, Freie Universität Berlin, Koenigin-Luise-Str. 2+4, 14195 Berlin, Germany; E-Mail: JaneckiA@rki.de (A.J.)

**Keywords:** flavanols, proanthocyanidins, anti-adhesion, group A-streptococci

## Abstract

Bacterial adhesion to epithelial cells is a key step in infections, allowing subsequent colonization, invasion and internalization of pathogens into tissues. Anti-adhesive agents are therefore potential prophylactic tools against bacterial infections. The range of anti-adhesive compounds is largely confined to carbohydrate analogues. Tannins are generously recognized as potent antimicrobials, but little data exist on their anti-adherence potency. Using a model for mucosal pathogenesis with labeled group A-streptococci (GAS) and human laryngeal HEp-2 cells, a series of flavan-3-ols (epicatechin, epigallocatechin, epigallocatechin-3-O-gallate) and highly purified and chemically characterized proanthocyanidin samples including procyanidins based on epicatechin, catechin or ‘mixed’ constituent flavanyl units, prodelphinidins made up of (epi)gallocatechin monomeric unts as well as oligomers possessing A-type units in their molecules was evaluated for anti-adhesive effects. Reduced microbial adherence was observed exclusively for prodelphinidins, suggesting that pyrogallol-type elements, *i.e.*, (epi)gallocatechin units are important structural features. This is the first report on structure-activity relationships regarding the anti-adhesive potency of proanthocyanidins. In addition, the structures of the first chemically defined proanthocyanidins from *Pelargonium sidoides* are disclosed.

## 1. Introduction

Proanthocyanidins represent a major group of polyphenols with amazing structural diversity that occur ubiquitously in woody and some herbaceous plants. Their presence in significant amounts in the wood, bark, leaves and fruits of many plant species has suggested a crucial role at the ecological and evolutionary level [1,2]. One of their well-documented functions includes protecting plants against herbivores. Proanthocyanidins are also considered as remarkable antimicrobials and potent inhibitors of viral infections in many ecological systems and in a number of *in vitro* experiments. Although there is good evidence for the adaptation of some herbivores and microbes to this constitutive chemical defense, plants may have benefited from accumulating large quantities of polyphenols in their tissues.

The recorded uses of traditional herbal medicines rich in proanthocanidins as effective antiseptic drugs may therefore appear only reasonable. The mode of action of these polyphenols is generally attributed to polyphenol-protein interactions, though different mechanisms have been suggested including inhibition of microbial enzymes, action on membranes or deprivation of substrates required for microbial growth [3]. Conspicuously, evaluation of the antimicrobial potency of a series of chemically defined tannins against a panel of microorganisms revealed only moderate growth inhibition, with minimum inhibitory concentrations (MIC) of 1,000 µg/mL for most samples [4]. This apparent discrepancy between weak antimicrobial activities of distinct phenolic compounds *in vitro *and effective antimicrobial protection to plants as well as the claimed efficacy of herbal medicines may plausibly be explained by the large polyphenol concentrations in plants and traditional herbal preparations as well as the chemical heterogeneity. From a clinical point of view, it is highly unlikely that under therapeutic conditions antimicrobial activity at such high MIC values will be significantly effective. Furthermore, the use of high quantities of tannins is therapeutically inappropriate, suggesting that indirect mechanisms may represent a conceivable alternative. Stimulation of the non-specific immune system by ingested polyphenolic metabolites present in herbal preparations may well provide a scientific basis for claimed remedial effects in infectious conditions. That proanthocyanidins have been shown to possess NO inducing and cytokine producing capabilities in infected macrophages strongly support this assumption [5,6]. Furthermore, inhibition of adhesion at an early stage would be effective in protecting host cells from infection. Adhesion of pathogenic bacteria to host cell surface is a crucial event in colonization and infection. Only little information is available on the anti-adhesive potentials of proanthocyanidins, largely confined to reports on cranberry for the prevention of urinary tact infections [7,8]. 

The hitherto limited information on the anti-adherence activities of proanthocyanidins in the interaction between pathogens and host epithelia prompted the present study. The paper presents results regarding the relationship between some flavan-3-ol/proanthocyanidin structures and adherence inhibitor activity in a model using group A-streptococci and human laryngeal epithelial (HEp-2) cells. 

## 2. Results and Discussion

The adhesion of pathogenic bacteria to epithelial cells is the first step towards development of an infection. This docking process is mediated by specific adhesions located on the outer microbial cell wall or on fimbriae [9]. Interestingly, most of the receptor-ligand interactions are carbohydrate-mediated systems. Thus, it appears only reasonable that much attention has been paid to carbohydrates as anti-adhesive agents of potential medicinal value that block the surface adhesions. Bearing in mind the well-known capabilities of proanthocyanidins to interact with macromolecules, including carbohydrates and proteins, members of this class of compounds may be another group of promising anti-adhesive compounds. Although broadly considered a non-specific process, previous work has provided evidence for the preference of proanthocyanidins for proline-rich proteins, dependency on the conformation of reactants, pH values and concentration, suggesting at least some kind of selectivity [10,11,12]. At present there is no report on proanthocyanidins as anti-adhesives concerning structure-activity relationships. Due to extreme difficulties in obtaining distinct proanthocyanidins in their free phenolic forms and availability of only some specimens in low quantities, highly purified and chemically defined oligomeric fractions were used for testing. 

**Figure 1:**
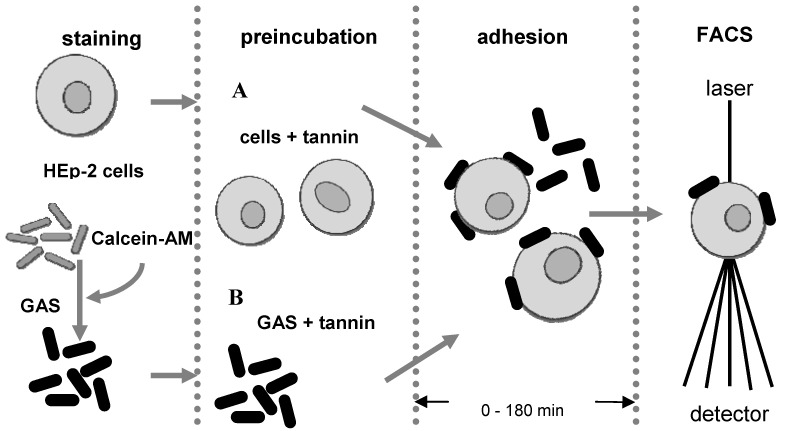
Flow cytometry-based anti-adhesive assay.

In a FACS-based assay [13], fluorescent-labelled group A-streptococci (GAS) were incubated with human epithelial cells. While GAS are commonly associated with pharyngitis and have been shown to invade epithelial cells [14], HEp-2 cells originate from the larynx. This experimental design provides an excellent model for mucosal pathogenesis with some therapeutic relevance in the treatment with herbal medicines containing proanthocyanidins. For investigation of a potential anti-adhesive effect, a set of co-incubation experiments was performed after pre-treatment of either the bacteria or the epithelial cells with the proanthocyanidin samples at a concentration of 30 µg/mL (Figure 1). Conspicuously, reduced microbial adhesion to HEp-2 cells was only apparent upon pre-treatment of GAS with the proanthocyanidin samples (data of pre-treated HEp-2 cells are therefore not shown). This finding clearly indicates that the interaction of the anti-adhesive principle occurred only with the bacterial outer membrane surface and not with binding sides at the epithelial surface. It should be noted that the number of colony forming units in non-treated and sample-treated bacteria were very similar, indicating that a possible antibacterial effect of the test substances was not responsible for the reduced bacterial attachment.

### 2.1. Anti-adhesive potency of flavan-3-ols

**Figure 2:**
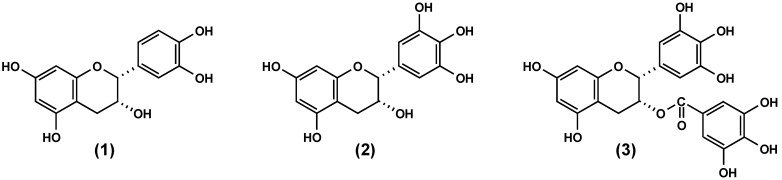
Structures of flavan-3-ols.

**Figure 3:**
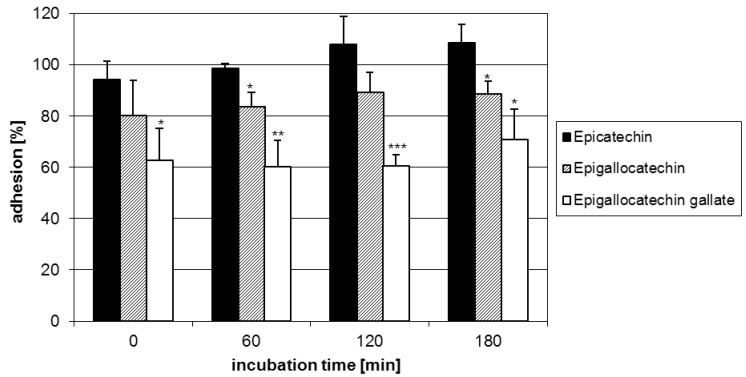
Adhesion of GAS pre-treated with the flavan-3-ols EC (**1**), EGC (**2**) and EGCG (**3**) to human HEp-2 cells as assessed by FACS analysis.Data represent the mean ± SD of epithelial cells that showed fluorescent bacteria and are derived from at least three independent experiments (* p < 0.05; ** p< 0.01; *** p< 0.001).

Starting with the anti-adhesive activity of flavan-3-ols, a series of 2,3-*cis-*configured analogues including (-)-epicatechin (**1**), (-)-epigallocatechin (**2**) and (-)-epigallocatechin-3-O-gallate (**3**) was evaluated. Compound **3** exhibited the highest relative reduction (ca. 40%) in microbial adhesion over the time of measurements, followed by **2** (ca. 15%), while **1** showed only negligible effects (Figure 3). The anti-adhesive potency of flavan-3-ols is apparently associated with the presence of pyrogallol-type elements as evident from the activity of **2** possessing a trihydroxylated B-ring and **3** with an additional galloyl group in their molecules. As shown in Figure 3, the antiadhesive activity increased well with the number of these structural features. In this context, it is appropriate to note that the arrangement of hydroxyl functions rather than their number is an appropriate structural determinant (*vide infra*). Although not yet tested due to non-availability, a similar behaviour in the anti-adhesive activity may be anticipated for 2,3-*trans* analogues. Figure 2.

### 2.2. Anti-adhesive potency of proanthocyanidins: structure-activity relationships

**Figure 4:**
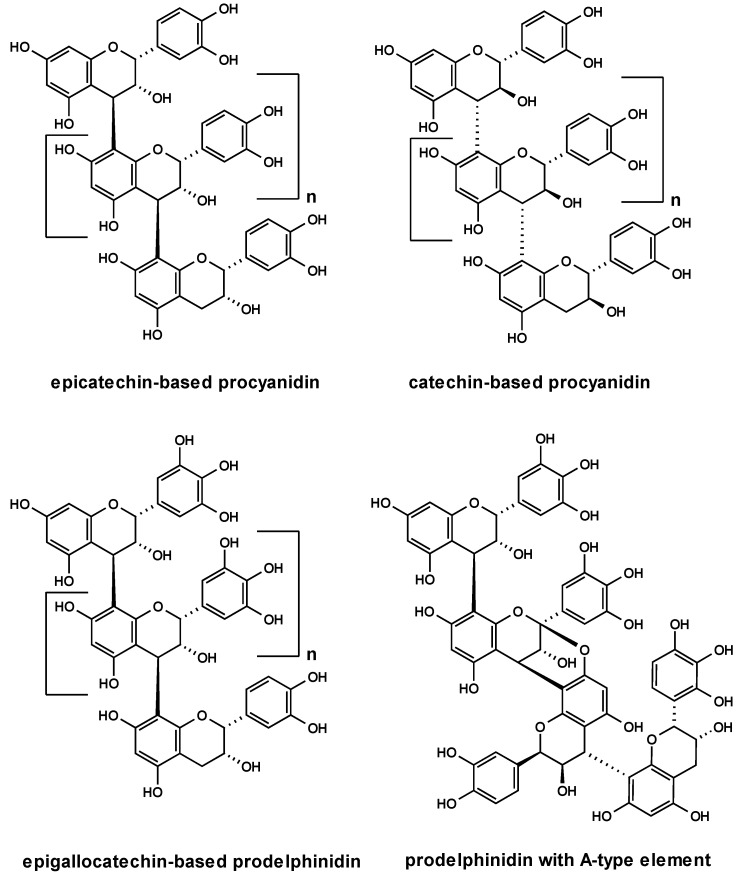
Structures of oligomeric proanthocyanidins.

Encouraged by the above findings, we extended our studies to a series of highly purified and chemically defined proanthocyanidin samples including epicatechin-based procyanidins from *Nelia meyeri* [15], catechin-based oligomers from *Salix *spp. [16], ‘mixed’-procyanidins from *Betula* spp. [17], prodelphinidins from *Ginkgo biloba* [18] and prodelphinidins with A-type elements from *Pelargonium sidoides* [19] (Figure 4). The percentage of proanthocyanidins was 100% in each instance, except for the sample of *P. sidoides* that was in the range of 40%. Although these specimens represented mixtures of oligomers, their principle constituent flavanyl units and the structures of a few components of each sample have been firmly established. The picture that emerged from this study did not necessarily imply the testing of distinct oligomers. It should be noted that homogeneous oligomers are difficult to obtain due to the polydisperse character of tannin compositions. 

**Figure 5:**
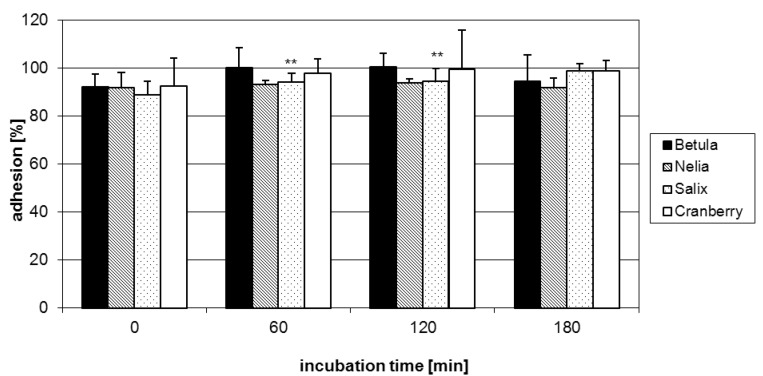
Adhesion of GAS pre-treated with purified A- and B-type procyanidin samples to human HEp-2 cells as assessed by FACS analysis. Data represent the mean ± SD of epithelial cells that showed fluorescent bacteria and are derived from at least three independent experiments (* p < 0.05).

Following the well-documented anti-adhesive effects of *Vaccinium macrocarpon* fruits [7,8], it appeared reasonable to include a cranberry extract in our experiments. Several A-type proanthocyanidins have been identified as the putative anti-adhesive principle for prophylaxis of urinary tract infections [7,20,21]. Adherence of pathogenic *E. coli *is accomplished by binding of lectins exposed on the surface of fimbriae to complementary carbohydrates on the host tissue [22]. In particular, P-fimbriae associated with the α-galactose-(1→4)-β-galactose specific lectin attach to the galabiose-containing structures on the uroepithelial cell surface [23]. The documented reduction in P-fimbriated *E. coli* adhesion may be rationalized by the close structural similarity of A-type units with their uncommon additional ether linkage between flavanyl constituent units and the galabiose lectin entity. Conspicuously, the tested cranberry extract did not show any anti-adhesive activity in our experimental model (Figure 5), indicating that the presence of A-type units represented no important structural feature in the interaction of group A-streptococci and HEp-2 cells. Regarding the model microorganism in the current work, fibronectin- and collagen-binding adhesions have been suggested to play an important role in the binding of *S. pyogenes* to host cells [24]. It appears therefore reasonable to conclude different types of docking processes. 

An additional structural feature examined included the nature of monomeric building blocks with emphasis on the hydroxylation patterns on the B-ring, *i.e.* (epi)catechin and (epi)gallocatechin entities. Evaluation of the range of procyanidin samples revealed that these molecules in all the examined stereochemical variants lacked anti-adhesive properties (Figure 5). This finding suggested that sources of B-type procyanidins did not represent promising anti-adhesion agents.

**Figure 6:**
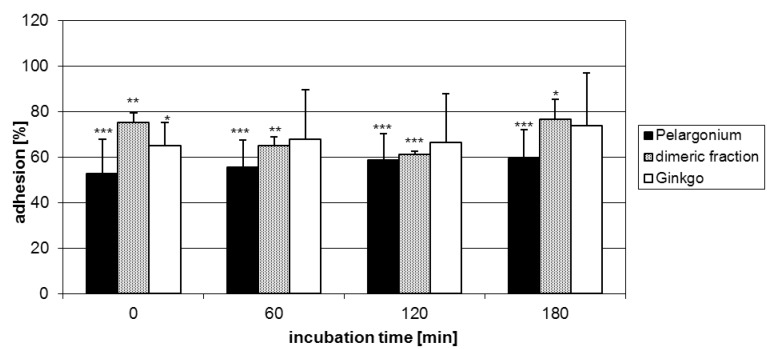
Effect of prodelphinidin samples on the adhesion of calcein-AM stained GAS to human HEp-2 cells as assessed by FACS analysis. Data represent the mean ± SD of epithelial cells that showed fluorescent bacteria and are derived from at least three independent experiments (* p < 0.05; ** p < 0.01; *** p < 0.001).

Next, the impact of prodelphinidins based on epigallocatechin/gallocatechin constituent flavanyl units represented by extracts of *G. biloba* and *P. sidoides* on the adhesion of GAS to host HEp-2 cells was tested. The samples showed pronounced anti-adhesive activities as evident from a ca. 35-45% reduced microbial adherence (Figure 6). This finding provided evidence that anti-adhesion of proanthocyanidins is apparently strongly associated with the presence of (epi)gallocatechin constituent units, at least for GAS. The degree of polymerization associated with an increase in the number of pyrogallol-type elements may have an effect on the anti-adherence potency. Conspicuously, the anti-adhesion capacity of the oligomeric samples was similar to that of the monomer (-)-epigallocatechin-3-O-gallate (**3**) (ca. 40%), but significantly more potent than that of (-)-epigallocatechin (**2**) (ca. 20%). Additional information was anticipated from a dimeric prodelphinidin fraction obtained from *P. sidoides*. Interestingly, this sample showed similar potentials, at least at the time points of 60 min and 120 min, when compared with the parent extract (Figure 6). Taking into account that this sample comprised a proportion of procyanidin substructures (see Section 2.5) shown to be inactive, the degree of condensation appears to be a minor contributing factor to this particular biological activity. A minimum of two pyrogallol-type elements may be concluded as necessary structural determinants for reasonable inhibition of GAS adherence to laryngeal epithelial cells.

### 2.3. Characterization of proanthocyanidins from Pelargonium sidoides

Although *P. sidoides* is known to contain large amounts of proanthocyanidins, structures of defined molecules have hitherto not yet been reported [25]. It appears therefore appropriate to briefly report on the structural assessment of the first chemically defined proanthocyanidins from this plant source. The quantities of samples did not permit the testing of their antiadhesive potentials. Multiple chromatography of the root extract on Sephadex LH-20 and elution with EtOH afforded a range of oligomeric fractions. The presence of gallocatechin and epigallocatechin in earlier test tubes was verified by HPLC-MS analysis. Examination of a subsequent eluted fraction revealed a mixture of procyanidin and prodelphinidin dimers, as evident from the ion peaks (MALDI-TOF) at *m/z* 595 and 611, respectively. While the latter mass data suggested two (epi)gallocatechin constituent units, the difference of 16 mass units in the former indicated that one of these units was replaced by (epi)catechin. Supporting evidence was available from acid hydrolysis of the mixture, producing delphinidin and cyanidin respectively. Further purification of the dimeric fraction, achieved by HPLC on RP-18 material, identified four proanthocyanidins hitherto unknown for the title species. Their structures were established on the basis of acid hydrolysis, MS analysis and examination of the ^1^H, ^13^C NMR, COSY and HMBC spectra. 

**Figure 7:**
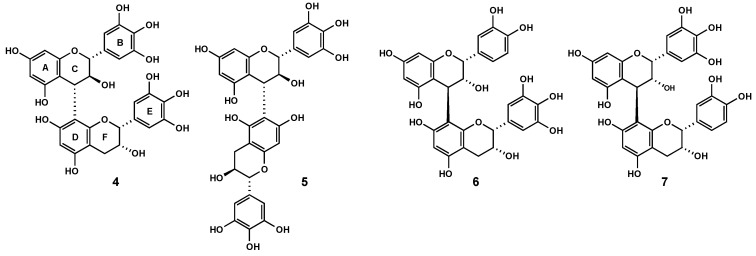
Structures of proanthocyanidins **4**-**7**.

The ^1^H-NMR spectrum of the major compound **4 **displayed two sharp low field proton singlets at δ 6.59 and δ 6.56, respectively, indicating that the B- and E-rings had the pyrogallol hydroxylation pattern. From a ^1^H-^1^H COSY spectrum the heterocyclic spin systems of the C and F rings were readily assigned. The coupling constants (*J_2,3 _*= 7.9 Hz, *J_3,4_* = 9.7 Hz) established the relative 2,3-*tran*-3,4-*trans* configuration of the upper constituent unit, while the 2,3-*cis* stereochemistry of the lower unit was evident from the small coupling constant (*J_2,3_* = 4.3 Hz) and the rather narrow envelope of signals for the C-4 (F) methylene protons (δ 2.8 – 2.9). Independent support for a terminating flavan-3-ol unit with a 2,3-*cis* configuration was obtained from the ^13^C resonances of C-2 (F) and C-3 (F) at δ 79.9 and 67.4, respectively. The key correlations between C-8_a_ (D) (δ_C_ 155.3) and both H-4 (C) (δ 4.63) and H-2 (F) (δ 4.88) in the HMBC spectrum unequivocally proved the 4,8-interflavanyl linkage [26]. The absence of a characteristic upfield shift (γ-effect) of the C-2 (C) resonance (δ_C_ 84.0) indicated that the 4-aryl substituent adopted a quasiequatorial orientation [27]. Collectively, these data defined **4** as gallocatechin-(4α,8)-epigallocatechin, previously reported from *Ribes* species [28,29], Oolong tea [30] and *Trifolium repens* [31]. Figure 7.

A similar strategy led to the identification of the known proanthocyanidins gallocatechin-(4α,6)-gallocatechin (**5**) [32], epicatechin-(4ß,8)-epigallocatechin (**6**) [33] and the tentatively identified new epigallocatechin-(4ß,8)-epicatechin (**7**), obtained in extremely low quantities. A common feature of their ^1^H-NMR spectra was the partial duplication of signals due to dynamic rotational isomerism and extremely broad and overlapping absorptions associated with the presence of 2,3-*cis* configurated constituent flavanyl units. 

## 3. Experimental Section

### 3.1. Test substances and plant extracts

(-)-Epicatechin was purchased from Roth (Germany), while (-)-epicatechin-3-O-gallate, (-)-epigallocatechin and (-)-epigallocatechin-3-O-gallate were generous gifts from Prof. Daneel Ferreira, University of Mississippi (Oxford, MS, USA). Highly purified proanthocyanidin fractions obtained from *Nelia meyeri*, *Salix spp.* and *Betula spp *were available in our research group. The work-up procedure and the characterization of the oligomeric proanthocyanidin mixtures are fully described elsewhere [15,16,17]. The root extract of *Pelargonium sidoides* (EPs® 7630, an aqueous-ethanolic (11% m/m) extract), a dimeric proanthocyanidin fraction thereof, an aqueous extract of *Vaccinium macrocarpon *fruits, and a methanolic fraction of *Ginkgo biloba* leaves (EGb 761) obtained from successive chromatography (H_2_O, EtOH, MeOH) over Sephadex LH-20 were kindly provided by Dr. Willmar Schwabe Pharmaceuticals (Karlsruhe, Germany). Stock solutions (1 mg/mL) in sterile water were prepared and kept at 4 ºC until use. 

### 3.2. Bacterial strain and culture conditions

*Streptococcus pyogenes* DSM 2071 (serogroup A) were maintained on Columbia blood agar plates (Heipha, Germany) at 37 ºC. Two to three colonies were inoculated into vials containing 5 mL tryptone soy broth (Oxoid, England) and incubated for 24 h at 37 ºC. The bacterial suspension was centrifuged at 4.000 × g for 5 min, the supernatant discarded and the pellet washed three times with PBS (Biochrom, Germany). For staining, 5 µL of a stock solution (1.25 µg/mL DMSO) of calcein-acetoxymethylester (Invitrogen, Germany) was added to 10^9^ bacteria and incubated on a thermomixer (Eppendorf, Germany) at a frequency of 1100 revolutions × min^-1^ and 37 ºC for 50 min. Bacteria were then harvested and washed three times with PBS. The number of colony forming units was determined by plating serial dilutions on Columbia blood agar and counting individual colonies after incubation for 24 h at 37 ºC.

### 3.3. Human laryngeal epithelial cells (HEp-2 cells)

Human HEp-2 cells, available at the University Medical Center Freiburg (Germany), were cultured to confluence in Dulbecco’s modified Eagle’s medium (Biochrom, Germany) containing 10% fetal calf serum (PAA, Germany). Before further preparation, cells were rinsed once with PBS and detached from the flasks with 0.05% trypsin/0.02% EDTA.

### 3.4. Anti-adhesion assay

Adhesion of group A-steptococci to host HEp-2 epithelial cells was evaluated by a modified flow cytometric assay [13]. Briefly, calcein-stained bacteria (2.5 × 10^7^ colony forming units per mL) and HEp-2 cells (2.5 × 10^5 ^mL) were co-incubated on a thermomixer at a frequency of 700 revolutions × min^-1^ at 37 ºC. For incubating with the samples, bacteria were pre-treated with 30 µg/mL of the test sample for 60 min at 37 ºC and washed prior assay measurements and in parallel epithelial cells were similarly pre-treated with the samples. Adhesion kinetics were performed by analysis at 0, 30, 60, 120 and 180 min of co-incubation of calcein-AM stained GAS and HEp-2 cells. Maximal adhesion, measured in the untreated control groups (negative control), was defined as 100% adhesion.

### 3.5. Flow cytometry

Samples were measured with a FACScan flow cytometer (Becton Dickinson, Germany). The flow cytometry data were analysed using CellQuest Pro software. The epithelial cell population was gated and adhesion was determined by the percentage of epithelial cells that became fluorescent after attachment of stained bacteria. 

### 3.6. Statistical analysis

The statistical analyses were performed using Microcal OriginPro® 7.0 software for Windows®. The values represent means ± SD of at least three independent experiments. For adhesion assessment at a single time point a one sample *t*-test was applied.

### 3.7. Isolation of proanthocyanidins from P. sidoides

The extraction and isolation of the proanthocyanidins followed the procedure recently described [19]. Final purification of compounds **4** – **7** was achieved by HPLC on RP-18 material (5 µm; 8 × 250 mm) using a water-MeOH gradient system (1:0 → 0:1; 40 min, flow rate 4 mL/min). Since NMR data recorded in MeOH-d_6_ were not previously reported, these are included for comparative purposes. The spectra were recorded on a Bruker DRX-400 instrument. Chemical shifts are given in δ (ppm) and *J* values in Hz. 

*Gallocatechin-(4α,8)-epigallocatechin* (**4**). ^1^H-NMR (400 MHz): δ 2.8-2.9 [m, 4- H_2_ (F)], 4.21 [m, 3-H (F), 4.34 [d, *J *= 9.7 Hz, (2-H (C)], 4.55 [dd, *J* = 7.9 and 9.7 Hz, 3-H(C)], 4.63 [d, *J* = 7.9 Hz, 4-H(C)], 4.87 [br s, 2-H (F)], 5.78 [d, *J* = 2.4 Hz, 8-H(A)], 5.84 [d, *J* = 2.4 Hz, 6-H(A)], 5.98 [s, 6-H (D)], 6.56 and 6.59 [each s, 2’/6’-H (B and E)]. ^13^C-NMR (100 MHz): δ_C_ 29.8 [C-4 (F)], 38.9 [C-4 (C)], 67.4 [C-3 (F)], 73.7 [C-3 (C)], 79.9 [C-2 (F)], 84.0 [C-2 (C)], 96.2 [C-6 (A)], 97.5 [C-8 (A)], 97.7 [C-8 (D)], 99.4 [C-4a (D)], 106.7 [C-4a (C)], 107.2 and 107.7 [C-2 and C-6 (B and E)], 108.5 [C-6 (D)], 131.4 and 131.7 [C-1 (B and E)], 133.4 [C-4 (B and E)], 146.7 [C-3 and C-5 (B and E)], 155.2-158.7 [ C-5 (A and D), C-7 (A and D), C-8a (A and D)]. 

*Gallocatechin-(4α,6)-gallocatechin* (**5**). ^1^H-NMR (400 MHz; *rotamer): δ 2.4-2.8 [m, 4- H_2_ (F)], 3.84 and 4.04* [m, 3-H (F), 4.18 [d, *J *= 9.6 Hz, 2-H (C)], 4.29* [d, *J* = 8.2 Hz, 2-H (C)], 4.38 [d, *J *= 7.8 Hz, 4-H(C)], 4.46* [d, *J* = 8.0 Hz, 4-H(C)], 4.52 [m, 3-H(C) and 3-H(C)*], 4.52 [d, *J *= 6.8 Hz, 2-H (F)], 4.80* [2-H (F), overlapped with solvent signal], 5.73 [d, *J* = 2.4 Hz, 8-H(A)], 5.80[d, *J* = 2.4 Hz, 6-H(A)], 5.84 and 5.86* [each d, *J *= 2.4 Hz, 6-H (A)], 5.92 and 6.05* [ each s, 8-H (D)], 6.04, 6.35, 6.49*, 6.52* [each s, 2’/6’-H (B and E)]. ^13^C-NMR (100 MHz): δ_C_ 28.0 [C-4 (F)], 38.6 [C-4 (C)], 68.5 [C-3 (F)], 73.7 [C-3 (C)], 82.8 [C-2 (F)], 84.3 [C-2 (C)], 96.3 [C-6 (A)], 96.9 [C-8 (A)], 97.5 [C-8 (D)], 100.5 [C-4a (D)], 107.1 [C-4a (C)], 108.4 and 108.8 [C-2 and C-6 (B and E)], 110.3 [C-6 (D)], 131.7 [C-1 (B and E)], 133.9 [C-4 (B and E)], 146.3 and 146.7 [C-3 and C-5 (B and E)], 154.8-158.8 [ C-5 (A and D), C-7 (A and D), C-8a (A and D)]. 

*Epicatechin-(4ß,8)-epigallocatechin* (**6**). ^1^H-NMR data were consistent with those reported [33]. ^13^ C-NMR (100 MHz): δ_C_ 29.9 [C-4 (F)], 37.1 [C-4 (C)], 66.8 [C-3 (F)], 73.5 [C-3 (C)], 77.1 [C-2 (C)], 79.7 [C-2 (F)], 96.1 [C-6 (A)], 96.6 [C-8 (A)], 97.4 [C-6 (D)], 100.6 [C-4a (D)], 107.2 [C-4a (C)], 108 - 110 [C-2, C-3, C-5, C-6 ( B and E)], 109.7 [C-8 (D)], 131.3 and 131.9 [C-1 (B and E)], 134.8 [C-4 (B and E)], 146.7 [C-3 and C-5 (B and E)], 156-159 [ C-5 (A and D), C-7 (A and D), C-8a (A and D)]. 

*Epigallocatechin-(4ß,8)-epicatechin* (**7**). ^1^H-NMR (400 MHz): δ 2.7-2.8 [m, 4- H_2_ (F)], 4.03 [m, 3-H (C)], 4.29 [3-H (C)], 4.79 [br s, 4-H(C)], 4.91 [br s, 2-H (F)], 5.07 [br s, 2-H (C)], 5.91-5.93 [br m, 6-H and 8-H (A)], 5.99 [br s, 6-H (D)], 6.82-6.90 [m, 5 H (B and E)].

## 4. Conclusions

Besides carbohydrates [34,35,36,37], proanthocyanidins may represent another group of promising anti-adhesion compounds. Previous studies were hitherto confined to *Vaccinium* polyphenols for the prophylaxis of urinary tract infections and, to some extent, to *Helicobacter pylori*-associated gastritis [7,38]. Apart from this, a single report on *Bridelia grandis* for microbial adhesion of streptococci suggests tannins as the anti-adhesive principle [39]. The blocking of adhesions in a broad spectrum of bacterial adherence may be rationalized by a kind of more or less specific proanthocyanidin-protein interaction. The limited information about the possible polyphenolic structures may suggest a non-specific process. Detailed investigations have provided evidence for some selectivity in tannin-protein interactions based on the conformation of reactants, pH values and the preference of polyphenols for proline-rich proteins [10,11,12]. The current data reveal important differences in the anti-adhesion activity between individual proanthocyanidins and suggest at least some selectivity toward particular biological targets in the process of bacterial adherence to host cells in this *in vitro* model. This study provided some insight into structure-activity relationships regarding the adhesive potential of proanthocyanidins. The question of specificity deserves further examination. On the other hand, information about the adhesion molecules would significantly contribute to a better understanding of the interactions.
